# Study on the Influence of Label Image Accuracy on the Performance of Concrete Crack Segmentation Network Models

**DOI:** 10.3390/s24041068

**Published:** 2024-02-06

**Authors:** Kaifeng Ma, Mengshu Hao, Wenlong Shang, Jinping Liu, Junzhen Meng, Qingfeng Hu, Peipei He, Shiming Li

**Affiliations:** College of Surveying and Geo-Informatics, North China University of Water Resources and Electric Power, Zhengzhou 450046, China; 201701425@stu.ncwu.edu.cn (M.H.); z20211151041@stu.ncwu.edu.cn (W.S.); liujp@radi.ac.cn (J.L.); mengjunzhen@ncwu.edu.cn (J.M.); huqingfeng@ncwu.edu.cn (Q.H.); hepei@ncwu.edu.cn (P.H.); lishiming@ncwu.edu.cn (S.L.)

**Keywords:** concrete crack segmentation, label image accuracy, DLNM, SSNM, accuracy evaluation

## Abstract

A high-quality dataset is a basic requirement to ensure the training quality and prediction accuracy of a deep learning network model (DLNM). To explore the influence of label image accuracy on the performance of a concrete crack segmentation network model in a semantic segmentation dataset, this study uses three labelling strategies, namely pixel-level fine labelling, outer contour widening labelling and topological structure widening labelling, respectively, to generate crack label images and construct three sets of crack semantic segmentation datasets with different accuracy. Four semantic segmentation network models (SSNMs), U-Net, High-Resolution Net (HRNet)V2, Pyramid Scene Parsing Network (PSPNet) and DeepLabV3+, were used for learning and training. The results show that the datasets constructed from the crack label images with pix-el-level fine labelling are more conducive to improving the accuracy of the network model for crack image segmentation. The U-Net had the best performance among the four SSNMs. The Mean Intersection over Union (MIoU), Mean Pixel Accuracy (MPA) and Accuracy reached 85.47%, 90.86% and 98.66%, respectively. The average difference between the quantized width of the crack image segmentation obtained by U-Net and the real crack width was 0.734 pixels, the maximum difference was 1.997 pixels, and the minimum difference was 0.141 pixels. Therefore, to improve the segmentation accuracy of crack images, the pixel-level fine labelling strategy and U-Net are the best choices.

## 1. Introduction

Concrete cracks are a common damage characteristic in engineering structures. They reflect the stress and damage state of engineering structures, which seriously affects the functionality and safety of engineering facilities. In order to ensure the safe and stable operation of engineering facilities, crack detection has become an important work content in the engineering quality supervision literature [1]. Traditional crack detection methods mainly rely on manual eye inspection, resulting in low detection efficiency, different detection standards, and difficult-to-guarantee accuracy [2]. Advances in computer vision technology have led to the emergence of image-based non-destructive testing techniques that can effectively complement the limitations of traditional manual inspection, which is time-consuming and subjective. These techniques include the minimal path selection method [3], the 3D multi-feature detection method [4], the K-means clustering segmentation method [5], etc. However, their manual design features and image quality requirements remain significant. As a result, the generalisation performance of the model is not strong [6]. With the rapid development of artificial intelligence technology, computer vision research based on deep learning has made many breakthroughs and has also brought forth many application scenarios for crack detection research. Compared with traditional image processing methods, the deep learning algorithm avoids complex image pre-processing. It adaptively learns and optimises the model without having to set too many parameters. In addition, deep features can be extracted from image data to effectively deal with cracks with different shapes and complex backgrounds [7]. At present, deep learning algorithms based on convolutional neural networks have been tried and achieved many results in the task of accurately locating and classifying cracks [8,9,10,11,12,13,14,15,16,17,18,19,20]. Among them, compared with the object detection network model, the crack detection algorithm based on SSNM can not only classify and locate the crack object but also obtain the pixel-level contour of the crack, so as to provide finer and higher-level semantic information for subsequent visual applications.

Due to the particularity of crack morphology and structure, the direct use of the mainstream SSNM for crack detection may not be satisfactory [9]. Therefore, Ma et al. [10] studied the effect of coupling the deep learning framework and convolutional neural network on the efficiency of crack detection, and some researchers tried to improve the detection performance of the network model for crack objects by improving the network structure. Zhu et al. [11] proposed the crack U-Net based on the U-Net model and adopted a dense connection structure to improve the utilization rate of the feature information of each layer in the network, so as to fully acquire the crack features and enhance the generalization ability of the network. The open crack datasets CRACK500 and CFD are used for model training and generalisation experiments, respectively. The results show that the proposed improved module can fully capture the crack characteristics and achieve the effect of improving the generalisation ability of the network. Liu et al. [12] improved the ability of the network to suppress various interference factors by embedding a parallel attention mechanism in the decoding part of the U-net. Due to the small size of the self-constructed dataset used, the performance of the improved network model has improved, but there is still room for improvement. Tan et al. [13] added the YOLOF module and the ResNet module based on DeepLabV3+ to improve the learning ability of the network model for crack feature information. The training results based on the self-constructed datasets containing 1290 bridge crack images showed that the improved network can significantly improve the segmentation accuracy of bridge crack images under different backgrounds. Huang et al. [14] reconstructed the ASPP module based on the DeepLabV3+ network using dense connections to improve the accuracy of crack detection under a complex background. However, the crack prediction results of the network model based on the public crack datasets CRACK500 and CFD still showed local fracture. Li et al. [15] introduced the spatial location module based on the self-attention mechanism into the PSPNet network and used the original datasets of 2000 bridge crack images to train the model. The trained network model achieved good segmentation results for small cracks and complex cracks. Zhang et al. [16] used lightweight MoblileNetv2 to replace ResNet, the original backbone feature extraction network of PSPNet, and introduced the position attention module to obtain rich contextual information, so that similar features at different positions could enhance each other and improve crack detection results. Zhong et al. [17] proposed a multi-scale feature fusion deep neural network structure w-SegNet based on the SegNet network, which has strong robustness for crack detection in various scenarios. Zheng et al. [18] proposed a high-precision crack detection method for lightweight concrete bridges based on SegNet and the separable convolutional residual of bottleneck depth, and they used 1500 self-collected bridge crack images for model training. Experiments showed that the improved network model could effectively eliminate the influence of background noise in crack images on the crack detection effect and improve the accuracy of the detection results. In Ref. [19], an improved dynamic feature fusion (DFF) network was used to achieve more accurate and clearer prediction of the edges of slender cracks by HRNet. Based on the HRNet network, He et al. [20] proposed an EJSNet model with high detection accuracy for crack detection by modifying the residual structure of the first stage, introducing the feature selection module (FSM) and receiving a field block (RFB) module, and improving the CBAM attention mechanism.

The above research provides many effective solutions for optimising the structure of the network model and improving the accuracy of crack detection and makes an important contribution to the realisation of automatic crack detection. However, another important factor that determines the performance of DLNM is somewhat neglected—the construction of the datasets. The essence of DLNM is to use a large number of datasets to extract and learn image features through supervised convolution operations, then minimise classification errors on training sets through backpropagation, optimise network parameters and finally achieve target extraction [21]. In the construction of deep learning algorithms, dataset preparation is crucial [22]. For SSNM, the model training dataset consists of two parts: the original image and the label image containing the contour mask of the object. As a guide for parameter optimisation of SSNM, the accuracy of the label images directly affects the learning efficiency of DLNM and further affects the segmentation accuracy of the trained model on the object [23].

In crack segmentation, because cracks are linear objects with topological structure and their widths change irregularly, coupled with the influence of the shooting distance during image acquisition, some cracks occupy a relatively small proportion in the image, making them difficult to identify with the naked eye; also, the production cost of label data is high, and the labelling accuracy is difficult to ensure. In order to explore the influence of crack image labelling accuracy on the performance of crack SSNMs, this paper takes the crack label image accuracy as the starting point, adopts three labelling strategies to make crack label images, and combines with the original crack image to construct three kinds of crack semantic segmentation datasets with different accuracy. In addition, four SSNMs, U-Net, HRNetV2, PSPNet and DeepLabV3+, were used for comparative experiments. By evaluating the accuracy of the trained network model and the accuracy of the crack image segmentation results, the optimal dataset construction method for crack semantic segmentation was obtained. It provides new ideas and schemes to improve the performance of the crack segmentation network model, and it also provides a practical reference for other similar crack detection projects.

## 2. Image Semantic Segmentation (ISS)

ISS is a technology that enables computers to automatically segment images and identify image content, and it is a combination of image classification, object detection and image segmentation technologies [24]. Due to limited computing power in days gone by, traditional ISS methods mainly rely on shallow visual features, such as image colour, texture and geometry, to segment image objects and then manually label semantic information to complete the image segmentation [25]. Some scholars proposed an active contour model based on local Kulback–Leibler divergence for fast image segmentation to overcome image inhomogeneity and noise, and it has a good segmentation effect in both real-world and medical images [26]. With the development of deep learning technology, ISS technology has entered a new era. At the 2015 IEEE International Conference on Computer Vision and Pattern Recognition, Ref. [27] proposed Fully Convolutional Networks (FCNs). FCNs solve the problem of repetitive storage and computational convolution caused by the use of pixel blocks in traditional segmentation networks, thus promoting the rapid development of ISS. However, there are still shortcomings, such as insensitivity to image details, failure to effectively consider image context feature information, complex training and large computation [28]. Therefore, based on FCN, researchers continue to extend and improve it and have proposed a series of more efficient ISS algorithms, such as U-Net, SegNet, PSPNet, RefineNet and DeepLab [29]. Among them, three representative improvement ideas are a method based on an encoder–decoder structure, a method based on feature fusion, and a method based on void convolution [30].

### 2.1. Method Based on Encoder–Decoder Structure

**Figure 1:**
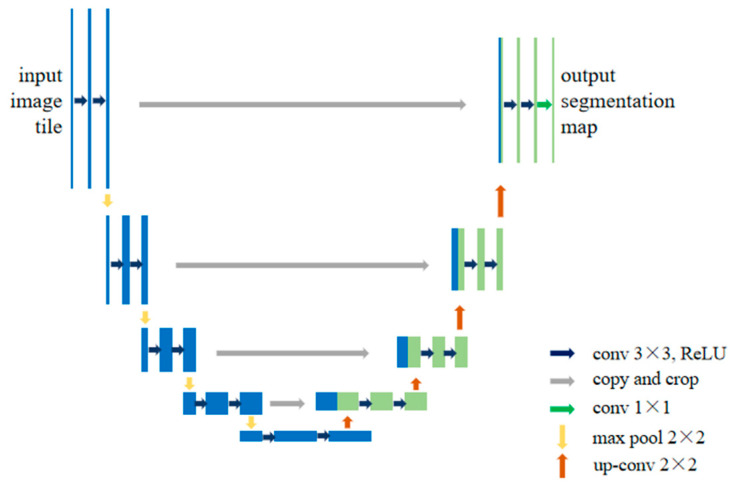
U-Net structure.

For problems such as image resolution reduction and pixel spatial information loss caused by the pooling operation, an effective method is to introduce an encoder–decoder structure [31]. U-Net is a classical encoder–decoder network proposed by Ronneberger et al. [32] in 2015. Its design was primarily intended for medical image segmentation, and due to its excellent performance, it has been gradually applied to various segmentation tasks [33]. The U-Net structure includes encoder, decoder and three same-layer skip connection components; the network structure is shown in Figure 1. The encoder consists of several convolution and pooling layers, which are used to obtain feature maps containing positional and semantic information from the original image. The decoder consists of a deconvolution layer and a pooling layer, whose function is to recover the lost spatial dimension and position information in the feature maps and to generate a dense prediction map. Another core idea of the network is to introduce skip connections, use feature splicing methods for feature fusion, make full use of the contextual information of the image and greatly improve the accuracy of image segmentation.

### 2.2. Method Based on Feature Fusion

**Figure 2:**
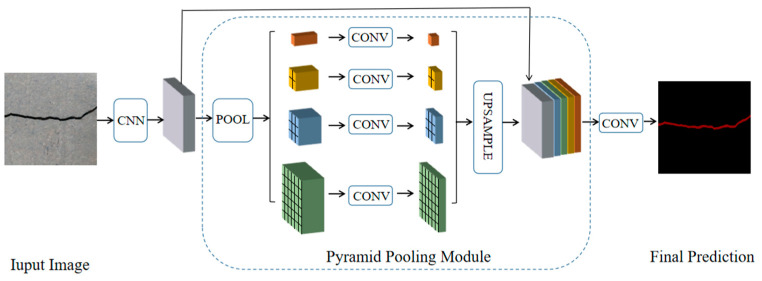
PSPNet structure.

FCN networks extract local features of images for pixel classification and do not use global features and context information, resulting in coarse segmentation results. To effectively solve the problem of connecting contextual information, Zhao et al. [34] proposed the PSPNet based on spatial pyramid pooling (SPP) in 2017, and the network structure is shown in Figure 2. The PSPNet mainly consists of three parts: the feature extraction module, the pyramid pooling module, and the output module. Among them, the pyramid pooling module of PSPNet obtains four feature maps of different levels by pooling the input features at four different scales, then upsamples the feature maps of different levels to recover the size before pooling and concatenates them with the features before pooling and finally generates the final prediction map via a convolution operation [16]. Based on the above structure, PSPNet can effectively use local and global contextual information by integrating features at different scales, thus effectively improving the speed and performance of ISS.

**Figure 3:**
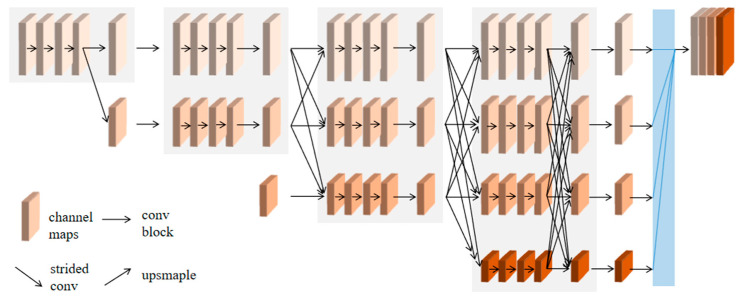
HRNet structure.

HRNet was proposed by the University of Science and Technology of China and the Microsoft Research Institute of Asia in 2019 [35]. It is primarily designed for human pose recognition. Later, HRNetV2 and HRNetV2p were proposed based on HRNet, and the HRNetV2 suitable for ISS is adopted in this paper. Its network structure is shown in Figure 3. Unlike the structure of most SSNMs, which first reduce the resolution and then increase the resolution, HRNetV2 connects high-resolution subnets to low-resolution subnets in parallel and adds the interaction between feature maps of different resolutions based on parallel by repeatedly exchanging information on parallel multi-resolution subnets to perform multi-scale repeated fusion. Each feature, from high to low resolution, repeatedly receives information from other parallel features, resulting in rich high-resolution features [36]. This network structure effectively reduces the downsampling effect caused by the use of step-length volume and pooling operations in the serially connected network model and narrows the semantic gap in information integration to some extent, so that the model’s ability to extract contextual features is significantly enhanced, and the accuracy of semantic segmentation is improved.

### 2.3. Method Based on Void Convolution

**Figure 4:**
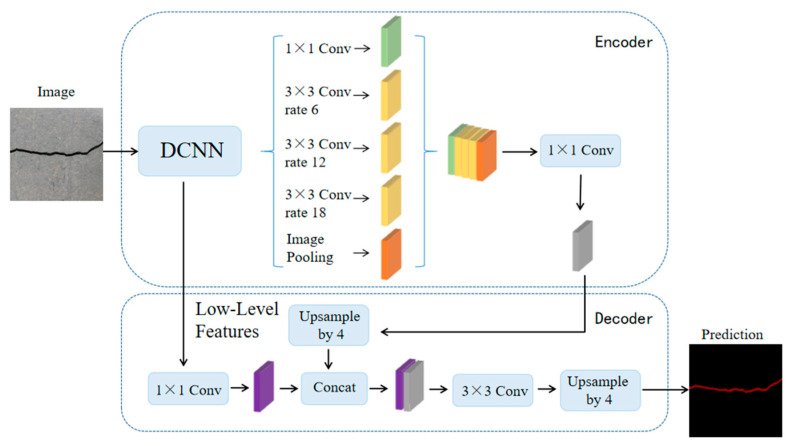
DeepLabV3+ structure.

Aiming to address problems such as the loss of partial spatial information and the lack of use of image context information caused by the smaller receptive field of the feature map in the FCN during downsampling, Chen et al. [37] proposed the DeepLabV1 in 2014. It creatively replaces part of the convolutional layer of the deep convolutional neural network (DCNN) with atrous convolution, which expands the receptive field without increasing the parameters, to obtain more feature information. Subsequently, based on DeepLabV1, the team successively proposed DeepLabV2, DeepLabV3 and other network models in the DeepLab series by continuously improving the use of atrous convolution and the network structure. DeepLabV3+, as the last network structure in the DeepLab series, is an excellent representative of the series model based on atrous convolution and multi-scale. Its network structure is shown in Figure 4. DeepLabV3+ uses DeepLab as the encoder of the network and adds a decoder module to recover the details of the object boundaries [38]. At the same time, depth-separable convolution is added to the ASPP and decoder modules to improve the speed and robustness of codec-structured networks.

Using DeepLabV3+ for ISS, the image processing is as follows: Input images first enter the DCNN at the coding end for feature extraction and output two types of feature maps. For the convolutional feature maps output at the last layer of the backbone network, they are sent to the ASPP module and used for multi-scale feature extraction with expansion convolution of different expansion rates to capture more contextual information and output advanced semantic features. For the shallow feature map output from the middle layer of the backbone network, it is input to the decoder and passed through the 1 × 1 convolution compression function. The decoder then performs a fourfold upsampling operation on the high-level semantic features output from the encoder and fuses them with the compressed shallow features of the same resolution. Finally, a prediction map with the same resolution as the original image is obtained by the linear interpolation upsampling operation.

## 3. Dataset Construction

### 3.1. Transfer Learning

Datasets are the source of signals that guide the DLNM to understand information. The data size, data diversity and data category distribution of the datasets have a large impact on the final performance of the algorithm [8]. To train a network model from scratch to achieve a preset effect, a huge amount of data are needed to support it. For people who do not have a large amount of data and computational resources, transfer learning is an effective strategy to improve the generalisation ability of neural network models [39].

Transfer learning makes full use of open-source data information, reduces the dependence of the object tasks on the amount of data and reduces training costs [40]. For the object network model, a large number of public datasets are used for pre-training so that the parameters of the network model reach a certain level of optimisation and then the weight obtained by pre-training is further adjusted on the dataset of a specific task so that the parameters of the network model are more suitable for the object task. In general, the source domain and the object domain with the highest possible similarity are prerequisites for ensuring the migration effect. However, for network models built for different computer vision tasks, although their goals and focus are different, the principle for extracting lower-level features is often similar, so the network can be pre-trained using public datasets and parameter sharing can be achieved to a certain extent.

In order to solve the problem of the small data size in the self-constructed concrete crack image dataset, the transfer learning training method is used in this paper to improve the generalisation ability of the crack segmentation network model. First, the model parameters are initialised on the source domain dataset ImageNet [41] to compensate for the lack of data in the self-constructed dataset and to improve the stability of the network model. The network model is then trained on the self-constructed crack dataset in the object domain, and the parameters of the pre-trained model are fine-tuned to make the network model more suitable for crack detection.

### 3.2. Construction of Datasets with Different Labelling Accuracy

**Figure 5:**
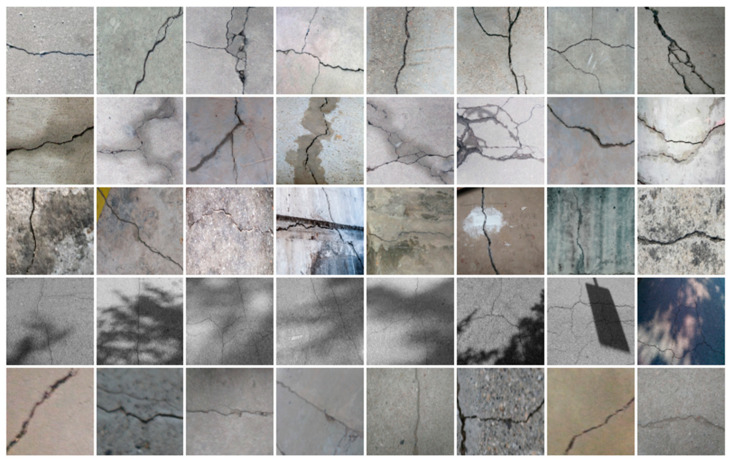
Example images of concrete crack datasets.

Two datasets are required to train the crack segmentation network model based on transfer learning. One is the source domain ImageNet open dataset [41], which contains a variety of data types and is widely used in computer vision for pre-training the transfer learning model. The categories of the images it contains are similar to those in the object domain, which ensures a certain transfer effect. The other is the object domain concrete crack dataset. By downloading the public crack image dataset Concrete Crack Conglomerate Dataset [42], Labeled Cracks in the Wild (LCW) Dataset [43,44,45,46,47,48,49], a total of 11,918 original crack images were collected, including about 500 reticulated crack images. The constructed datasets include a large number of concrete crack images with various interferences, such as water stains, spots, shadows, blur, etc., so that the network model can learn rich crack features and improve the detection robustness of the network model to crack images with complex background noise to some extent. Figure 5 shows an example of the types of crack images contained in the concrete crack datasets.

**Figure 6:**
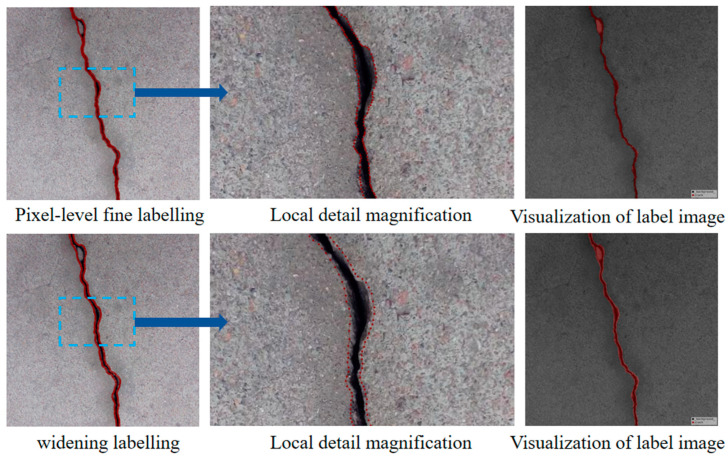
Crack image labelling process.

The image data labelling tool, labelme, was used to label the crack images according to two label categories: background and crack. Each crack image uses three labelling strategies: pixel-level fine labelling, outer contour widening labelling and topological structure widening labelling. This means that each original crack image corresponds to three crack label images. Among them, the unilateral deviation of the crack label image with fine labelling is generally controlled within 0.5 to 1 pixel, while the unilateral deviation of the crack label image with widened labelling is controlled within 1 to 2.5 pixels. Figure 6 shows a detailed comparison of the process of making the crack label image with the pixel-level fine labelling strategy and the widened labelling strategy, respectively.

Because the background of the concrete crack image is grey, the contrast between the crack and the background is low. The crack area is relatively small in the image, and there are many interfering factors such as stains and shadows that make labelling difficult. Among them, the outer contour widening labelling strategy does not need to be extremely close to the edge of the crack to accurately depict its zigzag contour features and the internal topology of the reticulated crack, so the cost and difficulty of labelling are relatively low among the three labelling strategies. Although the topological structure widening labelling strategy does not need to be extremely close to the edge of the crack to accurately depict its sawtooth contour features, it does need to consider and depict the internal topological contour of the reticulated crack. Therefore, the time cost of generating the entire dataset is approximately one-twentieth higher than that of the outer contour widening labelling strategy. As the most accurate labelling method, the pixel-level fine labelling strategy not only needs to accurately depict the outer zigzag contour features extremely close to the crack edge but also needs to accurately depict the inner topological contour of the reticulated crack. Therefore, the time required to produce the entire dataset is approximately 1.5-times that of the outer contour widening labelling strategy. 

Three groups of crack semantic segmentation datasets were constructed by combining the original crack image with the corresponding three types of label images, and the training, verification and test sets were divided according to a ratio of 8:1:1. According to the basic morphology and damage degree, the cracks can be divided into four categories, such as fine cracks, strong cracks, cross cracks and reticulated cracks [49]. According to the crack categories, the crack label images generated by three labelling strategies are shown in Table 1.

## 4. Evaluation Methods of Network Model Performance

### 4.1. Network Model Accuracy Evaluation Methods

**Figure 7:**
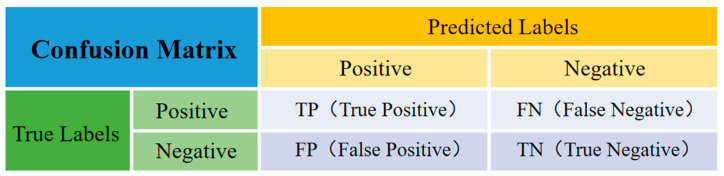
Confusion matrix of the crack detection network model.

The essence of crack detection is to distinguish the crack from the background information, which is the standard binary classification. If the crack is recorded as positive, the background as negative, the result of correct model prediction is recorded as true and the result of incorrect prediction is recorded as false, then based on the above four basic elements, a confusion matrix of the crack detection network model is established and shown in Figure 7. Based on the confusion matrix, higher-level model accuracy evaluation indicators, such as MIoU, MPA and Accuracy, can be obtained [50]. The definition and calculation methods of the evaluation indicators are shown in Table 2, where cracked pixels belong to the positive category and non-cracked pixels belong to the negative category.

### 4.2. Quantization Method of Crack Parameters

For the crack detection task, it is necessary to obtain not only the crack contour information but also the crack dimension parameters according to the segmentation results, provided that the semantic segmentation results match the real crack contour. As one of the key parameters in crack detection, crack width is also the most effective indicator to evaluate the segmentation accuracy of network models. Given the double-edge characteristics of the crack, the width of a crack can be defined as the Euclidean distance between the contour points closest to a skeleton point on either side of the edge, or the Euclidean distance between the orthogonal vector at a skeleton point and the intersection of the two edges of the crack. However, due to the pixel dispersion of the crack contour, a single quantization method based on the shortest distance or orthogonal idea will inevitably introduce quantization errors. In order to improve the accuracy of crack width calculation and ensure the reliability of network model segmentation accuracy evaluation, a minimum distance method with additional orthogonal constraints was proposed by synthesising the above two quantization ideas [51] to improve the accuracy and robustness of the crack quantization algorithm. 

**Figure 8:**
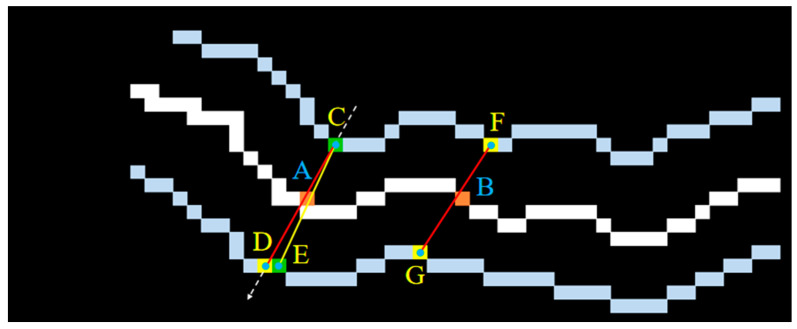
Schematic of crack width calculation using different quantification algorithms.

Figure 8 shows example plots of crack widths obtained by the shortest distance method, the orthogonal method and the shortest distance method with additional orthogonal constraints. Among them, the line segment CD represents the crack width at skeleton point A obtained by the orthogonal method, and the line segment FG represents the crack width at skeleton point B obtained by the shortest distance method. As can be seen from Figure 8, the line segment FG representing the crack width at skeleton point B, obtained by the shortest distance method, is severely distorted due to the jagged edges of the crack. The line segment CD representing the crack width at skeleton point A obtained by the orthogonal method is relatively accurate, but it does not inherently avoid the influence of the random mutation of the pixels at the crack edge, which leads to the uncontrollability of the accuracy of the crack width quantification results. The line segment CE is the crack width at skeleton point A obtained using the shortest distance method with additional orthogonal constraints. It can be seen that the crack width at skeleton point A represented by the line segment CE is more reasonable compared with the line segment CD.

(1) The crack contour is extracted using an edge detection algorithm, and then the skeleton of the crack image is extracted using an axis transformation or an image-thinning algorithm.
(2) The skeleton points are extracted sequentially on the crack skeleton line, and a 5 × 5 regional core is constructed with the skeleton points as the centre. The second-order moment of the connected domain composed of all the skeleton points in the regional core is used to calculate the crack extension direction *θ*, as shown in Equation (1) [52], and then the orthogonal vector of the crack extension direction is calculated using the orthogonal property.
  (1) $$\theta =\arctan\left[\frac{\left|\mu_{xx}-\mu_{yy}\right|+\sqrt{{\left(\mu_{xx}-\mu_{yy}\right)}^{2}-4\mu_{xy}^{2}}}{{2\mu }_{xy}}\right]$$
  where $\mu_{xx}={\sum_{i}^{N}\frac{\left(x_{i}-\overset{-}{x}\right)}{N}}^{2}+\frac{1}{12}$, $\mu_{yy}={\sum_{i}^{N}\frac{\left(y_{i}-\overset{-}{y}\right)}{N}}^{2}+\frac{1}{12}$, $\mu_{xy}=\sum_{i}^{N}\frac{x_{i}\cdot y_{i}}{N}$; where N is the total number of skeleton points in the region nucleus; $x_{i}$ and $y_{i}$ are the image coordinates of skeleton points in the region nucleus; $\overset{-}{x}$ and $\overset{-}{y}$ are the average value of the image coordinates of skeleton points.

**Figure 9:**
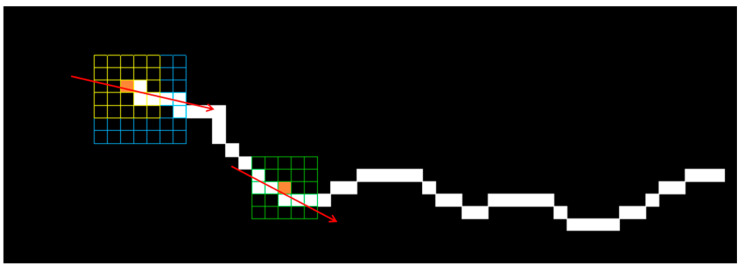
Schematic of calculating crack propagating direction.

Considering that there are two types of skeleton points, endpoint and non-endpoint, for skeleton points located at the non-endpoint, the 5 × 5 region can be directly overlayed on the skeleton diagram centred on the point of interest, as shown in the bottom right of Figure 9.

(1) With the skeleton point as the centre of the circle and a certain threshold as the radius, the search domain is set to obtain the local crack contour points for the skeleton point, and the projection coefficient of each local contour point onto the orthogonal vector is calculated. Due to the directivity of the vector, the projection coefficients of the contour points on both sides of the skeleton on the orthogonal vector are positive and negative. According to the positive and negative projection coefficients, the local contour points can be divided into two groups.
(2) The contribution coefficient *α* (0–1) is introduced to adjust the degree of contribution of the two quantization ideas to the crack width calculation results. The closer *α* is to 1, the greater the contribution of the orthogonal ideas.
(3) For a group with a positive projection coefficient, if the ratio of the local contour point’s projection coefficient to the maximum projection coefficient exceeds the contribution coefficient *α*, the contour point is considered as an alternative point and stored in set A. For a group with a negative projection coefficient, if the ratio of the local contour point’s projection coefficient to the minimum projection coefficient exceeds the contribution coefficient *α*, the contour point is considered as a candidate point and stored in set B. Finally, the combination with the shortest Euclidean distance between the two groups of candidate points is selected as the width of the crack at the skeleton point, and the width is calculated as shown in Equation (2).

(2) $$Width=\sqrt{{\left(x_{b}-x_{a}\right)}^{2}+{\left(y_{b}-y_{a}\right)}^{2}}$$

where $x_{a}$ and $y_{a}$ are the image coordinates of the candidate points in set A; $x_{b}$ and $y_{b}$ are the image coordinates of the candidate points in set B.

## 5. Experiments and Results

### 5.1. Experimental Environment and Parameter Settings

The hardware environment used in this experiment is as follows: the CPU is Intel (R) Core (TM) i7-8700 K CPU @ 3.70 GHz, the memory is 64 G, the graphics card model is NVIDIA GeForce RTX 2080 and the graphics memory is 8 G. The network model is built using Pytorch, a deep learning framework. Anaconda is used to configure the virtual Python environment needed to train the model, and CUDA and CUDNN are used to accelerate GPU computing, thus improving the training speed of the network.

The procedure for setting the hyperparameters of the network training is as follows: the stochastic gradient descent (SGD) algorithm is used to optimise the network model, the momentum parameter is set to 0.9 and the weight decay is set to 1 × 10^−4^. The network is trained for a total of 100 epochs, and the training batch size is 4. The maximum learning rate of the network is 1 × 10^−2^, the minimum learning rate is 0.01 of the maximum learning rate and the cos decreasing strategy is used to attenuate the learning rate.

### 5.2. Experimental Process

**Figure 10:**
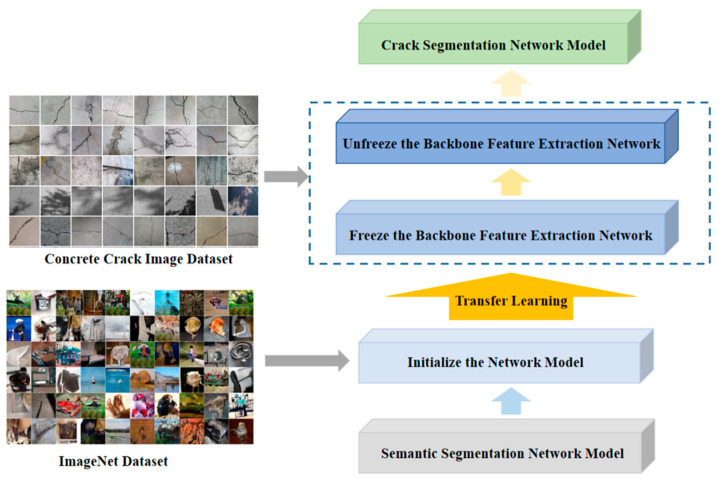
Training flow of crack segmentation network model based on transfer learning.

In this study, transfer learning was used to train the network model. First, the SSNM was trained on the source domain open dataset ImageNet to obtain the pre-training parameters of the network model. Then, the backbone feature extraction network of the SSNM was frozen, and the pre-trained model parameters were fine-tuned on the concrete crack dataset in the object domain to accelerate the model training speed and ensure the feature extraction effect and model stability. Finally, the backbone feature extraction network of the SSNM was unfrozen, and the feature extraction network parameters were retrained on the concrete crack dataset to improve the accuracy and generalisation ability of the SSNM; thus, the trained crack segmentation network model was obtained. The specific process is shown in Figure 10.

### 5.3. Comparative Experiment

#### 5.3.1. Accuracy Evaluation of Network Models

In order to facilitate analysis and expression, the dataset constructed in Section 3.2 is given a concise representation; that is, “dataset 1” represents “the dataset constructed by the crack original images + the label images labelled with pixel-level fineness”, “dataset 2” represents “the dataset constructed by the crack original images + the label images labelled with outer contour widening” and “dataset 3” represents “the dataset constructed by the crack original images + the label images labelled with topological structure widening”. According to the dataset constructed in Section 3.2, the four SSNMs presented in Section 2, U-Net, HRNetV2, PSPNet and DeepLabV3+, were used for training, and the evaluation indicators introduced in Section 4.1 were used to evaluate the accuracy of the trained network model and the results are shown in Table 3.

It can be seen from Table 3 that the network model is trained based on three sets of datasets, and the constructed dataset 1 has higher requirements for the learning ability of the network model. The network model trained based on dataset 1 has the lowest MIoU and MPA values, among which the PSPNet has the lowest MIoU and MPA values among the four SSNMs, which are 70.45% and 73.53%, respectively. However, the Accuracy value obtained by the four SSNMs is the highest, and the Accuracy value of the U-Net is the highest among the four SSNMs, reaching 98.88%. Using the constructed dataset 2 to train the network models, the PSPNet obtained higher MIoU and MPA values than the training results of the other two datasets, with values of 78.17% and 85.25%, respectively. The DeepLabV3+ obtained higher MPA values than the training results of the other two datasets, with values of 82.39%. However, the accuracy values obtained by the four SSNMs are all the lowest, with the DeepLabV3+ having the lowest accuracy value of 97.31% among the four SSNMs. The constructed dataset 3 was used to train the network models. With the exception of the PSPNet, the MIoU values of the other three SSNMs were higher, with the U-Net having the highest MIoU value of 85.47%. The U-Net has the highest MPA value of 90.86% among the four SSNMs. The Accuracy value of the four SSNMs is between the training results of the other two datasets. In summary, from the performance of the network models, the U-Net has the highest network stability and accuracy among the four SSNMs.

The segmentation results of the four SSNMs trained using the three constructed datasets for fine cracks, strong cracks and reticulated cracks are shown in Table 4, Table 5 and Table 6, respectively.

In terms of segmentation effect, only the U-Net trained by the constructed dataset 1 can segment the crack region more accurately for the three types of crack images, while the HRNetV2, PSPNet and DeepLabV3+ have a poor segmentation effect for the three types of crack images. The network model trained with the constructed dataset 2 shows good segmentation performance for fine and strong cracks in the four networks. For the reticulated crack, the U-Net has the same effect as the label images, and the segmentation effect of HRNetV2 is consistent with the topological structure of the crack. The crack contour segmented by PSPNet and DeepLabV3+ has a filling phenomenon inside, but the filling is incomplete and can easily be considered a misdetection. Using the network model trained by the constructed dataset 3, the four SSNMs achieve the best segmentation effect for the three types of crack images.

#### 5.3.2. Network Model Segmentation Accuracy Evaluation

From Section 5.3.1 it can be seen that the U-Net achieves better results than the other three SSNMs at several evaluation angles. Therefore, the U-Net is selected to further investigate the influence of the label image accuracy on the segmentation accuracy of the trained network models. To ensure the universality of the conclusion, crack images with different widths were selected for the comparative experiments. Table 7 shows the segmentation effect of the U-Net trained using three sets of constructed datasets for cracks of different widths. The actual crack contours are obtained using the image data labelling tool labelme, and the quantization values of the crack contours are calculated using the quantization algorithm proposed in Section 4.2; the results are marked in Table 7. MW is the maximum width, and AW is the average width.

Table 8 shows the difference between the segmented crack contour calculated by the U-Net trained with label images of different accuracy and the real crack contour. According to the analysis of the calculated results, in the crack segmentation results of the U-Net trained by the constructed dataset 1, the total average difference between the quantized result of the crack image segmentation contour and the real value is 0.734 pixels, the maximum difference is 1.997 pixels and the minimum difference is 0.141 pixels. In the crack segmentation results of the U-Net trained by the constructed dataset 2, the total average difference between the quantized results of the crack image segmentation contour and the real value is 8.86 pixels, the maximum difference is 11.537 pixels and the minimum difference is 5.941 pixels. In the crack segmentation results of the U-Net trained by the constructed dataset 3, the total average difference between the quantized result of the crack image segmentation contour and the real value is 8.06 pixels, the maximum difference is 10.302 pixels and the minimum is 5.260 pixels. Therefore, the comparative analysis of the experiment shows that the network model trained by the constructed dataset 1 has the most accurate segmentation results for the crack image, and the network model trained by the constructed dataset 2 has relatively poor segmentation results for the crack image, while the network model trained by the constructed dataset 3 has a large difference between the segmentation results of the crack image and the real contour. The experimental results provide useful guidance for the annotation construction strategy of the datasets.

## 6. Discussion

Due to the powerful feature extraction capability of deep learning algorithms, ISS algorithms based on convolutional neural networks have been widely used and continuously introduced. In order to reduce the dependence on pixel-level labelling samples in the training stage of network models, some scholars proposed to use weakly supervised learning for ISS and to use image-level labelling, border-level labelling, doodle labelling and other labelling methods to produce weakly labelled label images for network training [53,54,55]. Although the ISS method based on weak supervised learning has significant advantages in reducing the cost of training data labelling and speeding up the training data preparation process, for the object segmentation task of concrete cracks, weakly labelled images contain too little guide information compared to pixel-level labelled images, which cannot accurately describe the zigzag contour information of cracks and complex topological features. This is not conducive to improving the segmentation performance of the network model and anti-interference. However, manually labelled pixel-level image samples can provide a large amount of detailed information and local features, which is more conducive to improving the training efficiency and segmentation accuracy of the crack segmentation network model [56,57]. Gong et al. [58] proposed the concept of dataset life cycle and summarised it into five stages: dataset collection, dataset labelling, dataset storage, dataset testing and dataset destruction. Dataset labelling is a key process for transforming raw data into machine-identifiable information, and the quality of labelling is an important factor affecting the performance of machine learning models. In image labelling, the quality of the labelling depends on the accuracy of the label contour around the object, and the smaller the pixel distance between the label contour and the object, the higher the quality of the labelling. Therefore, for the crack label images generated by the three labelling strategies used in the experiment in this paper, the accuracy of the pixel-level fine labelling data is the highest, the accuracy of the outer contour widening labelling data is the lowest and the accuracy of the topological structure widening labelling data is in between. 

According to the evaluation results of the network model training accuracy, the accuracy of each SSNM shows different results after training with different accuracy levels of datasets. The Accuracy values of the four SSNMs trained with the pixel-level fine-labelled image data are higher than the results of the other two labelled datasets, among which the Accuracy value of the U-Net is as high as 98.88%. As the Ground Truth of the crack label images made with the pixel-level fine labelling strategy contains relatively few pixels, the pixel error tolerance of the predicted results is low, resulting in the lowest MIoU and MPA values. The MIoU values of the four SSNMs trained with image data labelled with topological structure widening, except PSPNet, U-Net, HRNetV2 and DeepLabV3+, were all higher than the results obtained from the other two labelled datasets, among which U-Net had the highest MIoU value, reaching 85.47%. The U-Net had the highest MPA value of 90.86% among the four SSNMs, and the Accuracy value of the four SSNMs was between the training results of the other two datasets. The Accuracy values of the four SSNMs trained on the image data labelled with outer contour widening were all lower than the results obtained from the other two labelled datasets, with the Accuracy value of DeepLabV3+ being the lowest at 97.31%.

It can be seen that in order to improve the accuracy of the crack segmentation network model, the crack label image must be made strictly according to the topological structure of the crack itself. According to the comparison results of the segmentation accuracy of the network model, it can be seen that the segmentation accuracy of the crack segmentation network model trained by datasets of different accuracy levels is consistent with the accuracy of the adopted datasets. In the crack segmentation network model based on the U-Net, the crack segmentation accuracy is highest in the model trained with image data labelled with a fine pixel-level, second in the model trained with image data labelled with topological structure widening and lowest in the model trained with image data labelled with outer contour widening. Among them, the U-Net trained with the pixel-level fine-labelled image dataset 1 has the highest data accuracy level, for the segmentation results of crack images, and the minimum difference between the quantized value of the segmentation contour width and the real width value of the crack is only 0.141 pixels and the maximum is no more than 2 pixels. The U-Net is trained using the image dataset 3 labelled with topological structure widening; for the segmentation results of crack images, the minimum difference between the quantized value of the segmentation contour width and the real width value of the crack is 5.260 pixels, and the maximum difference is 10.302 pixels. The U-Net is trained using the image dataset 2 labelled with the outer contour widening; for the segmentation results of the crack images, the minimum difference between the quantized value of the segmentation contour width and the real width value of the crack is 5.941 pixels, and the maximum difference is 11.537 pixels. 

This is because the model adjusts the weights during the training process by comparing the differences between the predicted results and the real labels to gradually improve the accuracy of our understanding of semantic information. The pixel-level fine-labelled crack label image provides accurate crack boundary information, enabling the model to learn more specific crack features, improve the understanding of crack details and better adjust the weight to adapt to the real scene during training, so that crack edge information can be more accurately captured in the segmentation process. However, the crack label images using the topology widening label and the outer contour widening label provide inaccurate crack boundary information, which leads to the model paying too much attention to the surrounding background and insufficient learning of the crack boundary information, resulting in poor segmentation results. It can be seen that the labelling accuracy of the label image has a significant impact on the segmentation accuracy of the crack segmentation network model. In order to obtain the crack segmentation results with higher accuracy, the labelling accuracy of the label image must be improved as much as possible.

## 7. Conclusions

In order to explore the effect of crack image labelling accuracy on the performance of concrete crack SSNMs, in this study, three labelling strategies, namely pixel-level fine labelling, outer contour widening labelling and topological structure widening labelling, were used to generate crack label images, respectively, and then combined with the original crack images to construct three sets of concrete crack semantic segmentation datasets with different accuracy. Each dataset contained 11,918 crack images and their corresponding label images. Firstly, transfer learning was used to pre-train U-Net, HRNetV2, PSPNet and DeepLabV3+ on the ImageNet dataset, initialise the model parameters and then construct three sets of datasets with different accuracy for network training, so as to improve the detection accuracy of the network model on the crack object.

(1) The comparison results of the network model training accuracy show that due to the specificity of the crack object, the labelling accuracy of the crack label image has a different influence on the performance of the SSNMs, and different SSNMs have different sensitivity to crack label images with different accuracy. The Accuracy values of the four SSNMs trained using the pixel-level fine label image are all the highest, among which the Accuracy values of the U-Net are the highest, while the MIoU and MPA values are the lowest. The Accuracy values of the four SSNMs trained using the image data labelled with outer contour widening are all the lowest, among which DeepLabV3+ has the lowest Accuracy value. For the Accuracy values of the four SSNMs trained on the image data labelled with topological structure widening, the MIoU value of the three SSNMs except the PSPNetV2 is the highest, and the U-Net has the highest MPA value. It can be seen that the labelling accuracy of the crack label images strongly affects the learning efficiency and training accuracy of the SSNMs.
(2) According to the comparison results of the segmentation effect of the network model, among the four SSNMs trained with pixel-level finely labelled image data, U-Net achieves accurate segmentation results for crack images with different segmentation difficulties, such as fine crack, strong crack and reticulated crack, and the segmentation contour is the most detailed. HRNetV2, PSPNet and DeepLabV3+ have good segmentation performance only for strong cracks. Four kinds of SSNMs were obtained using image data labelled with outer contour widening, and the segmentation results of fine cracks and strong cracks are more accurate. For the reticulated crack, U-Net obtained the same features as the labelled images, HRNetV2 obtained the same features as the reticulated crack topology and PSPNet and DeepLabV3+ obtained the same features as the labelled images, but the internal filling was incomplete. The four SSNMs trained on the image data labelled with the topological structure widening obtain complete segmentation results for the three types of crack images, but the segmentation contours are all wider than the real crack contours. It can be seen that the characteristics of the image labels have a profound effect on the learning efficiency of the network models and the segmentation effect of the crack images. In addition, the U-Net has a stronger learning ability than PSPNet, HRNetV2 and DeepLabV3+ and can better learn the crack characteristics for crack label images with different label accuracy. The model has high accuracy and strong stability, which is more suitable for crack detection.
(3) Compared with the segmentation accuracy of the network model, it can be seen that the finely labelled crack label images have a higher demand on the learning ability of the SSNM, but the trained network model has a crack segmentation contour that is closer to the real crack contour for the segmentation results of the crack image. However, the widened labelled crack image has a slightly lower learning requirement for the SSNM. The network model trained with the label image data of such accuracy has an increased width of the crack segmentation contour compared to the real crack contour. The U-Net is obtained using the image data of the outer contour widening label; for the segmentation results of the crack image, the quantified value of the contour width is very different from the real width value of the crack, but the segmented contour has better integrity and intuitionism, which is conducive to the location and identification of the crack. It can be seen that in order to improve the efficiency of crack detection and obtain accurate crack segmentation results, excellent network models and pixel-level fine labelling data are indispensable.

Due to the complexity and specificity of the crack contours, the creation of the label images is very time consuming and labour intensive. In addition, most researchers focus more on algorithm improvement, ignoring the impact of dataset sample size and label image quality on model performance. This study mainly discussed the effect of image labelling quality on the performance of crack detection network models. According to the accuracy requirements of crack detection tasks and the experimental conclusions of this paper, relevant researchers can select appropriate image labelling strategies to construct datasets, thus saving the cost of dataset labelling on the premise of achieving the object effect. The influence of sample size, mixed labelling accuracy and other factors on the performance of the crack detection network model will be further demonstrated in future research.

## Figures and Tables

**Table 1:** Crack label images generated by three labelling strategies.

Labelling Strategy Crack Type	Original Crack Image	Pixel-Level Fine Labelling	Outer Contour Widening Labelling	Topological Structure Widening Labelling
Fine crack	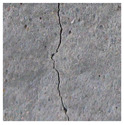	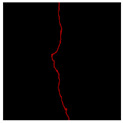	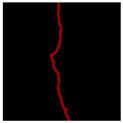	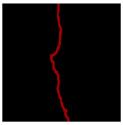
Strong crack	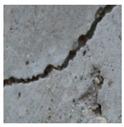	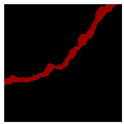	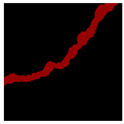	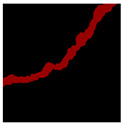
Cross crack	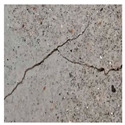	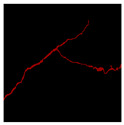	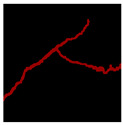	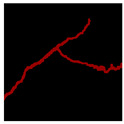
Reticulated crack	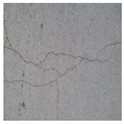	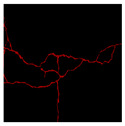	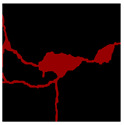	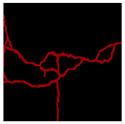

**Table 2:** Accuracy evaluation indicators of the network models.

Name	Definition	Instructions
TP	True Positive	The sample is predicted to be positive and the true label is positive.
FP	False Positive	The sample is predicted to be positive, but the true label is negative.
TN	True Negative	The sample is predicted to be negative and the true label is negative.
FN	False Negative	The sample is predicted to be negative, but the true label is positive.
MIou	Mean Intersection over Union	$\frac{1}{k+1}\sum_{i=0}^{k}\left(TP/\left(TP+FP+FN\right)\right)$ where k represents the number of categories.
MPA	Mean Pixel Accuracy	$\frac{1}{k+1}\sum_{i=0}^{k}\left(TP/\left(TP+FP\right)\right)$ where k represents the number of categories.
Accuracy	Pixel Accuracy	$\left(TP+TN\right)/\left(TP+FP+TN+FN\right)$

**Table 3:** Results of network model training accuracy evaluation.

Network Models	Training Datasets	Experimental Results		
		MioU (%)	MPA (%)	Accuracy (%)
U-Net	dataset 1	80.58	86.64	98.88
	dataset 2	83.52	89.41	98.17
	dataset 3	85.47	90.86	98.66
HRNetV2	dataset 1	71.59	75.33	98.42
	dataset 2	77.88	82.85	97.57
	dataset 3	78.06	82.94	97.97
PSPNet	dataset 1	70.45	73.53	98.40
	dataset 2	78.17	85.25	97.46
	dataset 3	77.33	82.71	97.87
DeepLabV3+	dataset 1	71.37	75.10	98.41
	dataset 2	76.36	82.39	97.31
	dataset 3	76.66	81.38	97.84

**Table 4:** The segmentation effect of four SSNM—fine cracks.

Training Datasets	Network Models			
	U-Net	HRNetV2	PSPNet	DeepLabV3+
dataset 1	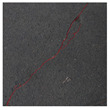	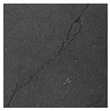	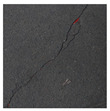	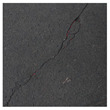
dataset 2	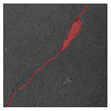	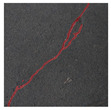	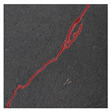	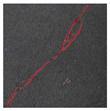
dataset 3	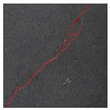	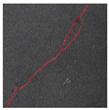	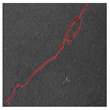	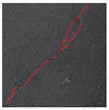

**Table 5:** The segmentation effect of four SSNMs—strong cracks.

Training Datasets	Network Models			
	U-Net	HRNetV2	PSPNet	DeepLabV3+
dataset 1	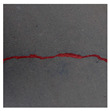	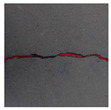	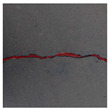	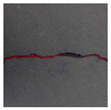
dataset 2	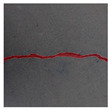	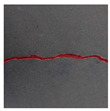	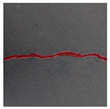	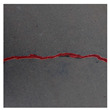
dataset 3	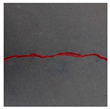	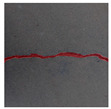	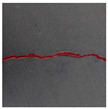	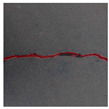

**Table 6:** The segmentation effect of four SSNMs—reticulated cracks.

Training Datasets	Network Models			
	U-Net	HRNetV2	PSPNet	DeepLabV3+
dataset 1	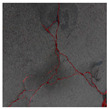	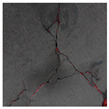	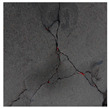	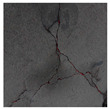
dataset 2	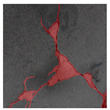	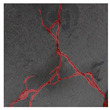	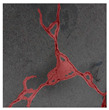	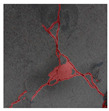
dataset 3	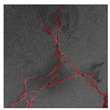	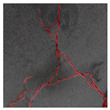	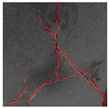	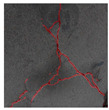

**Table 7:** Comparison of the crack segmentation effect of the U-Net trained with label images of different accuracy (unit: pixels).

Original Crack Image	Crack True Contour	Network Model Segmentation Contour		
		Dataset 1	Dataset 2	Dataset 3
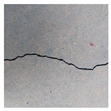	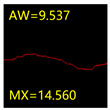	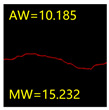	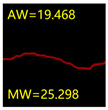	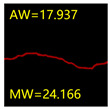
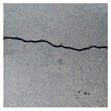	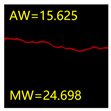	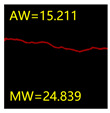	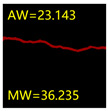	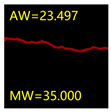
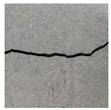	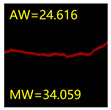	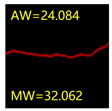	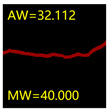	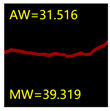

**Table 8:** Difference between the crack segmentation contour of the U-Net trained with label images of different accuracy and the real crack (unit: pixels).

Original Crack Image	Quantized Value	Dataset 1	Dataset 2	Dataset 3
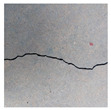	MW	+0.672	+10.738	+9.606
	AW	+0.648	+9.931	+8.400
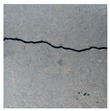	MW	+0.141	+11.537	+10.302
	AW	+0.414	+7.518	+7.872
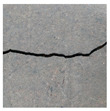	MW	+1.997	+5.941	+5.260
	AW	+0.532	+7.496	+6.900

## Data Availability

The data will be made available on request.
